# The activity of commercial antimicrobials, and essential oils and ethanolic extracts of *Olea europaea* on *Streptococcus agalactiae* isolated from pregnant women

**DOI:** 10.1186/s12906-019-2445-4

**Published:** 2019-01-30

**Authors:** Munyaradzi Mukesi, Benson C. Iweriebor, Larry C. Obi, Uchechukwu U. Nwodo, Sylvester R. Moyo, Anthony I. Okoh

**Affiliations:** 1 SAMRC Microbial Water Quality Monitoring Centre, University of Fort Hare, Private Bag X1314, Alice, Eastern Cape Province 5700 South Africa; 2 Applied and Environmental Microbiology Research Group (AEMREG), Department of Biochemistry and Microbiology, University of Fort Hare, Private Bag X1314, Alice, Eastern Cape Province 5700 South Africa; 3 Department of Health Sciences, Faculty of Health and Applied Sciences, Namibia University of Science and Technology, Private Bag 13388, Windhoek, Namibia; 4 Academic and Research Division, University of Fort Hare, Private Bag X1314, Alice, Eastern Cape Province 5700 South Africa

**Keywords:** *Olea europaea*, *Streptococcus agalactiae*, Antimicrobial, *β Lactams*

## Abstract

**Background:**

*Streptococcus agalactiae* also known as Group B Streptococcus (GBS) is a major cause of disease in pregnant women and new born babies where it causes early and late onset disease characterised by sepsis, pneumonia and meningitis. Ten to 37 % of pregnant women in the world are colonised with GBS while intrapartum antibiotic prophylaxis has led to significant reduction in early onset disease. The increase in drug resistant microorganisms has become a major threat. Development of vaccines is still in progress so there is need for new and safer alternatives to treatment.

**Methods:**

Benzyl penicillin, Ampicillin, Cefotaxime, Ceftriaxone, Levofloxacin, Erythromycin, Clindamycin, Linezolid, Vancomycin, Tetracycline and Cotrimoxazole, *Olea europaea* leaf extracts and essential oil were tested against GBS isolates from South Africa and Namibia.

**Results:**

The isolates showed 100% sensitivity to benzyl penicillin, ampicillin, ceftriaxone, levofloxacin, linezolid, vancomycin, *O. europaea* leaf extracts and essential oils. Only one isolate (0.6%) was resistant to cefotaxime and 23.4 and 10.4% were resistant to clindamycin and erythromycin respectively.

**Conclusion:**

GBS isolates showed sensitivity to *O. europaea* extracts at low minimum inhibitory concentrations. *Β lactams* are still the drugs of choice for treatment of GBS disease but *O. europaea* extracts potent as an alternative source of antimicrobials.

## Background

Antibiotic resistance has existed since the beginning of the antibiotic era. The increase in the frequency of drug resistant and invasive bacteria, a reduction in the development and approval of new drugs and projections of an imminent post antibiotic era threaten human health worldwide. Current practices of Intrapartum Antibiotic prophylaxis (IAP) against *S. agalactiae* involve the administration of β-lactam antibiotics penicillin and ampicillin. The alternatives to penicillin include: cefazolin, clindamycin, erythromycin and vancomycin however, these alternative drugs have not been assessed in clinical trials and data on their concentration levels in amniotic fluid, foetal blood and tissue are limited [1, 2].

There is an increasing prevalence of multidrug resistant microorganisms and these bacterial pathogens are a major threat to existing antibacterial therapeutic options [3]. GBS is generally sensitive to β-lactams antibiotics but isolates with reduced minimum inhibitory concentrations (MICs) have been described from as far back as 1995 [1] while MIC changes of cefazolin to GBS have been described from 1999 [2]. In a recent study in Italy, outright resistance to GBS was noted for the first time where out of 65 GBS isolates recovered in the study, 12.07% were resistant to benzyl penicillin [4].

Antibiotic resistance can arise due to the expression of genes that confer resistance to drugs in an organism. The most clinically significant genes are those that code for enzymes responsible for hydrolysis of β-lactams (*bla* genes). These genes confer high level bacterial resistance to β-lactam antibiotics like cephalosporins (including 4th generation cephalosporins), penicillins and monobactams (except cefamycins) [5]. The *bla* genes can be borne on plasmids or transposons which are transferable and this facilitates horizontal spread of antibiotic resistance [6].

While erythromycin and clindamycin were indicated as alternative therapy to GBS IAP, there has been increasing resistance of GBS to these drugs and in the United States of America (USA), clindamycin and erythromycin resistance have been recorded at 13–20% and 25–35% respectively [2]. Across the world, resistance has been estimated at 14.5–70% and 8.2–70% for erythromycin and clindamycin respectively [1]. The CDC guidelines removed erythromycin as a second line prophylactic antibiotic in women with penicillin allergy due to the high resistance of GBS to it while clindamycin is still acceptable but only after susceptibility testing [7].

The increasing resistance of GBS to empiric antibiotics has led to the administration of expensive drugs and those previously considered as very toxic [3]. While the development of vaccines is still in progress, there is need for new and safe alternatives to GBS treatment [1]. The World Health Organization (WHO) suggests the use of safe and effective medicinal plants; and in both developing and developed countries, herbal medicine has become an integral part of primary health care due to its low cost and availability [8]. The antimicrobial activities of several natural plant products against GBS that have been documented indicate them as potential alternatives to IAP. Essential oils from various plants have been shown to have antibacterial, antiviral, anti-leishmanial, antioxidant, analgesic and anti-inflammatory properties [1, 3]. One such plant which is widely used for its medicinal properties is *Olea europaea* species of the genus *Olea* and subspecies *Africana* which is known as umNquma in the Eastern Cape, South Africa.

*O. europaea* is known to contain a wide range of phytochemical components with medicinal properties from as far back as 1854 and a number of phytochemical components have been isolated from different parts of *O. europaea.* The bark, fruits, seeds, leaves, olive oil and wood contain phenolic compounds, flavonoids and secoridoids. Due to the presence of many compounds in different parts of the plant, it has an array of uses including control of plasma concentrations of glucose, cholesterol and urate [9].

Leaf extracts and essential oils from *O. europaea* have been reported to have antimicrobial activities against many microorganisms including bacteria, viruses, fungi and parasites. While research has shown that the plant does not have broad spectrum antimicrobial activity it has been shown to be potent against some Gram positive and Gram negative bacteria such as *Staphylococcus aureus* and *Escherichia coli* respectively [10]. Antimicrobial activity has also been shown against fungi such as *Candida albicans* and *Cryptococcus neoformans* while other researches have reported the antimicrobial activity of the plant against *Helicobacter pylori*, *Campylobacter jejuni*, *Bacillus subtilis* and *Pseudomonas aeruginosa* [9].

This study therefore documents the antibiotic susceptibility profiles of *S. agalactiae* isolates. It also seeks to determine the antimicrobial susceptibility of these isolates to leaf extracts and essential oils from *O. europaea* collected from the Cala community in the Eastern Cape, South Africa.

## Methods

### GBS culture, isolation and identification

A rectal and a vaginal swab were collected from each participant who were between 35 and 37 weeks gestation by a registered nurse. In South Africa (Eastern Cape), 100 participants were included in the study while in Namibia (Windhoek), 588 participants took part in the study. All swabs collected in South Africa were processed at the AEMREG laboratory, University of Fort Hare, Alice while samples in Namibia were processed at the Namibia University of Science and Technology, Microbiology Laboratory.

Samples were inoculated onto Columbia blood agar and Todd Hewitt broth (Rochelle Chemicals and Laboratory Equipment, Johannesburg, South Africa) and incubated at 37 °C for 24 h. Inoculums in Todd Hewitt broth were subcultured onto blood agar and incubated at 37 °C for 24 h and the cultures were checked for colonies exhibiting β-haemolysis on the blood agar. The colonies were tested for the catalase reaction and catalase negative colonies were further screened using the Lancefield grouping antisera (Becton Dickinson, New Jersey, USA). Colonies which tested positive for the latex agglutination assay were presumptively identified as GBS isolates and were confirmed using the Vitek 2 and by molecular techniques. All presumptive isolates were stored in glycerol stocks at − 80 °C until further analyses.

### Molecular confirmation of isolates

Glycerol stocks were inoculated in Muller Hinton broth for 24 h at 37 °C and streaked on Columbia blood agar. GBS isolates were confirmed by molecular techniques using a pair of primers specific for the *scp*B gene. A single colony of GBS was picked from Columbia blood agar containing 5% horse blood and suspended into 160 μL of nucleic acid free water in a 2 mL conical tube that was placed on a heating block and allowed to boil at 100 °C for 15 min. The mixture was centrifuged and the supernatant containing GBS DNA was transferd in a clean 1.5 mL microcentrifuge tube and stored at − 80 °C. The amplification reactions were performed using the One Taq® Master Mix (New England Biolabs, United Kingdom) according to the manufacturer’s recommendations. Thus, the reactions were performed in a final volume of 25 μL containing 12 μL of Master mix, 6 μL of purified DNA, 10 pmol of scpBF (5’ACAACGGAAGGCGCTACTGTTC3’) and scpBR (5’ACCTGGTGTTTGACCTGAACTA3’) primers as adapted from Elbaradie et al., 2009 [11] and deionized water to adjust the final volume.

The cycling conditions were as follows: an initial denaturation at 94 °C for 4 min followed by 35 cycles of: denaturation at 93 °C for 1 min, annealing at 57 °C for 1 min and extension at 72 °C for 1 min. A final elongation step of 72 °C for 7 min was followed by a hold at 4 °C. *S. agalactiae* ATCC reference strain BAA-2670 was used as positive control. Amplicons were loaded on 1% agarose gel stained with 10 μL ethidium bromide and electrophoresed for 45 mins at 110 V in 0.5X Tris/Borate/EDTA (TBE) buffer and viewed under transilluminator and photographed.

### Sensitivity testing using Vitek

Antimicrobial sensitivity testing was performed using Vitek Sensitivity Card (AST-ST01) on the following antibiotics: Benzyl penicillin, Ampicillin, Cefotaxime, Ceftriaxone, Levofloxacin, Erythromycin, Clindamycin, Linezolid, Vancomycin, Tetracycline, Cotrimoxazole and Inducible Clindamycin Resistance (ICR). MICs were determined for each antibiotic on all the isolates and the results were interpreted according to the Clinical Laboratory Standards Institute (CLSI, 2015) guidelines. The results were classified as sensitive (S), intermediate (I) or resistant (R). All isolates were evaluated for inducible clindamycin resistance.

### Plant material

*O. europaea* plant parts were collected in the Cala community, geographical coordinates 31.52.30°S, 27.69.80°E, Eastern Cape Province, South Africa during the winter of 2016. The plant was identified by Cala Traditional Health Practitioners using the vernacular name umNquma and was authenticated by Mr. Tony Dold, a Plant Taxonomist at Selmar Schonland Herbarium, Rhodes University, South Africa. The Voucher (No ADE 2016/2) was deposited in the Giffen Herbarium, University of Fort Hare.

### Preparation of plant extracts

Leaves were harvested from the plants and washed in water to remove dust. The leaves were rinsed in distilled water to cleanse them of impurities and were dried in air for 3 days. The leaves were pulverized as previously described by Darsanaki et al., 2012. Fifty grams of leaf powder was mixed with one litre of ethanol and placed on a shaker to solve and thereafter, the solution was filtered and the solvent dried to obtain a crude ethanol extract of the *O. europaea*. For the extraction of essential oils, 200 g of dried pulverized leaves was mixed with 200 mL of water and the mixture placed in a modified hydro distiller or a Clevenger’s type apparatus and allowed to hydro distillate for 3 h for complete extraction of the essential oils from the leaves. The procedure was repeated thrice to obtain adequate essential oil for experimental assays and the extracted essential oil was dried using anhydrous sodium sulphate and extracts stored at − 20 °C in actinic bottles from which working solutions were prepared with Dimethyl Sulfoxide (DMSO) for use in experiments.

### Gas chromatography-mass spectrometry (GC-MS) analysis

The GCMS analysis of essential oil was performed on the Agilent 5977A MSD and 7890B GC system, Chemetrix; Agilent Technologies, DE (Germany) with a Zibron-5MS column. GC grade helium at 2 mL/min flow rate and spitless 1 mL injection was used and temperatures were set at 280 °C (Injector), 280 °C (Source) and 70 °C (oven) while the ramp settings were set as follows and held for 3 min; 15 °C/min to 120 °C, followed by 10 °C/min to 180 °C and finally 20 °C/min to 270 °C.

### Preparation of plant extracts for antimicrobial activity

Plant extract and essential oils were diluted with 10% DMSO using serial dilution methods to make 300 mg/mL of essential oil and 0.5 mg/mL of leaf extract. These solutions were used as stock solutions for all antimicrobial testing.

### Agar well diffusion

One hundred microliters of 0.5 McFarland standard of each isolate was prepared using sterile normal saline (0.9% *w*/*v*). Twenty microliters of each 0.5 McFarland preparation was inoculated onto Mueller Hinton agar and spread for a lawn growth of the bacteria on the agar. Standard bores of eight millimetres in diameter were made in the agar plates using a sterile cork-borer. Ten percent DMSO was used as a negative control while 5 μg of ciprofloxacin served as a positive control. One hundred microliters each of the ethanolic leaf extracts (0.5 mg/ml), essential oils (300 mg/ml), positive control and negative control were put in labelled wells and allowed to stand for 1 h and culture plates incubated at 37 °C for 24 h. The test extracts were compared to the negative and positive control to determine the antimicrobial activity of the extracts on the isolates. Ten isolates each from both South Africa and Namibia were selected by systematic sampling and used in the experiment.

### Minimum inhibitory concentration (MIC) determination

Four hundred microliters of broth was put in each of four sterile tubes and serial dilutions were made using 100 μL of extract creating four dilutions of each extract. The final extract concentration in each tube for the extracts is presented in Table 1.

**Table 1:** Concentrations of plant extracts after dilution

Tube number	1	2	3	4
Concentration of essential oil (mg/ml)	33.3	3.7	0.4	0.05
Concentration of leaf extract (mg/ml)	0.1	0.02	0.004	0.0008

Broth was used as a negative control while 5 μg of ciprofloxacin was used as a positive control. One hundred microliters of broth was placed in six labelled tubes for each experiment. One hundred microliters each of broth (negative control), 5 μg ciprofloxacin (positive control) and each of the dilutions for both essential oil and plant extract were put in a respective labelled tube and 20 μL of 0.5 McFarland of the test isolate was inoculated in each tube and incubated at 37 °C for 24 h. Thereafter, the test tubes were checked for turbidity and compared to the negative and positive control to establish the antimicrobial activity of the different concentrations of the extracts. Tubes without apparent growth were regarded as concentrations where the extract had growth inhibitory activities.

For the concentrations of the extract which showed antimicrobial effect against the bacteria it was determined if the extract had bactericidal or bacteriostatic effect. This was performed by inoculating 20 μL from each of the tubes above onto Mueller Hinton agar and the culture plates were incubated at 37 °C for 24 h. Absence of growth in the broth and agar plate was assumed as the extract having bactericidal effect while absence of growth in broth and bacterial growth on solid agar plates, indicates bacteriostatic effect of the extract.

## Results

Results of antimicrobial sensitivity testing of antibiotics against GBS and susceptibility to plant extracts are presented below. Twenty GBS isolates (10 from South Africa and 10 from Namibia) were employed in antibacterial activities of testing of plant extracts using the Minimum Inhibitory Concentration (MIC) approach. The antibiotic sensitivity patterns of the GBS isolates are presented in Table 2.

**Table 2:** Antibiotic sensitivity patterns of GBS isolates

Antibiotic	MIC (μg/mL)	Frequency (%)		
		Susceptible	Intermediate	Resistant
Benzyl penicillin (P)	≤ 0.12	154 (100.0)	0 (0.0)	0 (0.0)
Ampicillin (Amp)	≤ 0.25	154 (100.0)	0 (0.0)	0 (0.0)
Cefotaxime (CTX)	≤ 0.5	153 (99.4)	0 (0.0)	1 (0.6)
Ceftriaxone (CRO)	≤ 0.5	154 (100.0)	0 (0.0)	0 (0.0)
Levofloxacin (Lev)	≤ 2	154 (100.0)	0 (0.0)	0 (0.0)
Erythromycin (E)	≤ 0.25	138 (89.6)	0 (0.0)	16 (10.4)
Clindamycin (CD)	≤ 0.25	118 (76.6)	0 (0.0)	36 (23.4)
Linezolid (Li)	≤ 2	154 (100.0)	0 (0.0)	0 (0.0)
Vancomycin (Va)	≤ 1	154 (100.0)	0 (0.0)	0 (0.0)
Tetracycline (T)	≤ 2	6 (3.9)	0 (0.0)	148 (96.1)
Cotrimoxazole (SXT)	≤ 2	137 (89.0)	0 (0.0)	17 (11.0)
Inducible clindamycin				7 (4.5)

*S. agalactiae* isolates were sensitive to B lactam antibiotics but some showed resistance to other antibiotics such as tetracycline, cotrimoxazole, erythromycin and clindamycin.

Table 3 shows the chemical composition of the essential oils of *Olea europaea* obtained after GC-MS analysis.

**Table 3:** Chemical composition of essential oils of *Olea europaea*

S/N	Chemical constituents	Chemical formula	RT	Area %
1	2-Butenal, 2-ethenyl-	C_6_H_8_O	3.255	1.56
2	2-Hexenal, (E)-	C_6_H_10_O	3.292	2.95
3	1-Hexanol	C_6_H_14_O	3.365	0.75
4	Heptanal	C_7_H_14_O	3.638	1.00
5	(1S)-2,6,6-trimethylbicyclo[3.1.1] hept-2-ene	C_10_H_16_	3.975	1.89
6	Pyridine, 3-ethenyl-	C_7_H_7_N	4.204	2.21
7	2,4-Heptadienal, (E,E)-	C_2_H_5_CH	4.412	1.58
8	Octanal	C_8_H_16_O	4.450	0.77
9	alpha-Phellandrene	C_10_H_16_	4.530	1.72
10	4-Carene	C_10_H_10_	4.729	0.79
11	2-Octenal, (E)-	C_8_H_14_O	4.908	0.50
12	1-Octanol	C_8_H_18_O	4.972	1.87
13	Pyridine, 5-ethenyl-2-methyl-	C_8_H_9_N	5.198	0.88
14	Nonanal	C_9_H_18_O	5.271	10.57
15	1,6-heptadiene,2,5,5-trimethyl	C_10_H_18_	5.674	0.73
16	Acetaldehyde, (3,3dimethylcyclohexylidene),(E)	C_10_H_18_O	5.720	1.07
17	1-Methylpentyl cyclopropane	C_9_H_18_	5.772	0.80
18	Decanal	C_10_H_20_O	6.059	2.23
19	3-Isopropylidene-5-methyl-hex-4-en-2-one	C_10_H_16_O	6.278	0.99
20	Octane, 1-iodo-	C_8_H_17_I	6.347	0.57
21	2,6-Octadien-1-ol, 3,7-dimethyl-(Z)-	C_10_H_18_O	6.418	0.39
22	2-Decenal, (E)-	C_10_H_18_O	6.485	2.78
23	2,4-Decadienal	C_10_H_16_O	6.724	0.76
24	4-tert-Butylcatechol, dimethyl ether	C_12_H_18_O_2_	6.809	4.63
25	9-Oxabicyclo [4.2.1] non-7-en-3-one	C_8_H_12_O	6.892	1.80
26	1-Oxaspiro [4.5] dec-6-ene, 2,6,10,10-tetramethyl-	C_13_H_22_O	6.995	1.10
27	2-Undecenal	C_11_H_20_O	7.206	0.78
28	cis-beta-Farnesene	C_15_H_24_	7.422	0.60
29	1-(3,6,6-trimethyl-1,6,7,7a-tetrahydrocyclopenta [c] pyran-1-yl) ethanone	C_13_H_18_	7.510	0.72
30	2-Buten-1-one,1-(2,6,6-trimethyl-1-cyclohexen-1-yl)-	C_13_H_20_O	7.631	0.50
31	Caryophyllene	C_15_H_24_	7.751	2.04
32	5,9-Undecadien-2-one, 6,10-dimethyl-	C_13_H_22_O	7.798	2.91
33	1H-Inden-1-one, 2,4,5,6,7,7a-hexahydro-4,4,7a-trimethyl-	C_12_H_18_O	7.931	0.46
34	1,4,7,-Cycloundecatriene, 1,5,9,9-tetramethyl-, Z,Z,Z-	C_15_H_24_	7.974	0.44
35	trans-beta-Ionone	C_13_H_20_O	8.093	2.39
36	21.xi-methyl-17-isocholest-16-en-3beta-ol	C_27_H_46_O	8.150	0.47
37	Naphthalene, 1,2,3,4,4a,5,6,8a-octahydro-4a,8-dimethyl-2- (1-methylethenyl)-,[2R-(2 alpha, 4a alpha, 8a beta)]	C_15_H_24_	8.232	0.69
38	Naphthalene, 1,2,4a,5,6,8a-hexahydro-4,7-dimethyl-1-(1-methylethyl)-	C_15_H_34_	8.323	2.91
39	1,6,10-Dodecatrien-3-ol,3711,trimethyl-, [S-(Z)]-	C_15_H_26_O	8.508	1.82
40	Benzoic acid, nonadecyl ester	C_26_H_44_O_2_	8.631	0.79
41	1H_Cycloprop[e] azulen-7-ol, decahydro-1,1,7-trimethyl-4-methylene-,[1ar-(1aalpha, 4a alpha, 7 beta, 7a beta, 7b alpha)]-	C_15_H_24_O	8.750	0.80
42	Caryophyllene	C_15_H_24O_	8.809	1.49
43	4,2,8-Ethanylylidene-2H-1-benzopyran, octahydro-4-methyl-	C_14_H_22_O	8.923	0.55
44	1,4-Methano-1H-indene, octahydro-1,7a-dimethyl-4-(1-methylethenyl)-[1S-(1 alpha, 3a beta, 4 alpha, 7a beta)]-	C_15_H_24_	8.961	0.52
45	Bicyclo[4.4.0]dec-1-ene, 2-isopropyl-5-methyl-9-methylene	C_15_H_24_	9.089	5.65
46	Heptadecane	C_17_H_36_	9.250	0.39
47	7-epi-cis-sesquisabinene hydrate	C_15_H_26_O	9.281	0.94
48	2,6,10-Dodecatrien-1-ol, 3,7,11,trimethyl	C_15_H_26_O	9.424	0.46
49	2-Pentadecanone, 6,10,14-trimethyl	C_18_H_36_O	10.058	1.66
50	1,2-Benzenedicarboxylic acid, bis(2-methylpropyl) ester	C_16_H_22_O_4_	10.235	0.72
51	Eicosane	C_20_H_42_	10.312	0.47
52	2,6,10,14,18-Pentamethyl-2,6,10,14,18-eicosapentaene	C_25_H_42_	10.467	0.97
53	Dibutyl phthalate	C_16_H_22_O_4_	10.713	0.43
54	Eicosane	C_20_H_42_	10.810	0.42
55	Isopropyl palmitate	C_19_H_38_O_2_	10.930	0.29
56	Heptasiloxane, 1,1,3,3,5,5,7,7,9,9,11,11,13,13-tetradecamethyl-	C_14_H_44_	11.235	0.09
57	Octadecane	C_18_H_38_	11.286	1.03
58	2-Myristynoyl-glycinamide	C_16_H_31_BrN_2_O_2_	11.336	0.77
59	Phytol	C_20_H_40_O	11.388	6.40
60	1H-Indole-2-carboxylic acid, 6-(4-ethoxyphenyl)-3-methyl-4-oxo-4,5,6,7-tetrahydro-, isopropyl ester	C_21_H_25_	11.741	0.55
61	Nonadecane	C_19_H_40_	12.174	1.09
62	Cyclotrisiloxane, hexamethyl	C_6_H_18_O_3_Si_3_	12.593	0.34
63	Heptasiloxane, 1,1,3,3,5,5,7,7,9,9,11,11,13,13-tetradecamethyl	C_14_H_44_O_6_Si_7_	12.994	0.98
64	Octasiloxane, 1,1,3,3,5,5,7,7,9,9,11,11,13,13,15,15-hexadecamethyl	C_16_H_48_O_7_Si_8_	13.779	1.81
65	Tetrasiloxane, decamethyl-	C_10_H_30_O_3_Si_4_	14.201	0.37
66	Silicic acid, diethyl bis (trimethylsilyl) ester	C_10_H_28_O_4_Si_3_	14.201	0.20
67	Heneicosane	C_21_H_44_	14.659	3.37
68	Tris (tert-butyldimethylsilyloxy) arsane	C_18_H_45_AsO_3_Si_3_	15.741	1.94

The GC-MS analysis of the plant extract showed that the plant contained flavonoids, flavonones, biophenols, benzoic acid derivatives, sterols and secoiridoids.

Essential oils and leaf extracts exhibited antimicrobial activity against GBS isolates and leaf extracts showed bigger zones of inhibition compared to essential oils.

## Discussion

In this study 117 isolates from Namibia and 37 isolates from South Africa were analyzed and they were confirmed as GBS based on the *scp*B gene. All the isolates showed absolute sensitivity to benzyl penicillin, ampicillin, ceftriaxone, levofloxacin, linezolid and vancomycin with only one isolate (0.6%) showing resistance to cefotaxime as shown in Table 2. In reports from other studies done in other African countries, such as in Malawi [12], Ethiopia [13], Zimbabwe [14], South Africa [15] and Nigeria [16], GBS has not shown resistance to the *β lactams*. In a study in Brazil, GBS also exhibited no resistance to *β lactams* [17]. However, resistance to *β lactam* group of antibiotics has been evolving as noted in reduced MICs for more than a decade and recently an Italian study recorded outright resistance by some GBS isolates to penicillin [4].

Thirty six isolates (23.4%) and sixteen isolates (10.4%) were resistant to clindamycin and erythromycin respectively and the results of this study are comparable to a report from a recent study in Pretoria, South Africa where GBS isolates had 17.2 and 21.16% resistance to clindamycin and erythromycin respectively [15]. The results also fall within world statistics of clindamycin resistance (8.2–70%) and erythromycin resistance (14.5–70%) [1]. However, studies in Brazil have recorded low GBS resistance to clindamycin (3.0%) and erythromycin (4.1%) [17].

There was high resistance of GBS to tetracycline (96.1%) and this appears consistent with other studies which have reported high GBS resistance to tetracycline (86.7%) in South Africa [15] and in Brazil [17] and this could be due to the indiscriminate use of tetracycline and its availability as a non-prescription drug. Tetracycline is commonly used at farms as a growth promoter and for treatment purposes. Resistance to the drug could spread through the environment due to agricultural runoff and contaminated effluent while antibiotic residue in farm produce like meat is another serious concern which perpetuates drug resistance as low levels of drugs in farm produce exert persistent selection pressure on bacteria thus giving rise to the emergence of resistant strains [18].

Resistance to cotrimoxazole was low in this study (11.0%) contrary to its reported high resistance level among GBS isolates from other studies in other African countries like Ethiopia (29.0%) [19]. In Tanzania, just like many other sub Saharan African countries burdened by human immunodeficiency virus (HIV) infection, cotrimoxazole is used for prophylactic purposes in all acquired immunodeficiency syndrome (AIDS) patients including pregnant women before 36 weeks gestation. Resistance to cotrimoxazole in Tanzania ranges from 56.0% in *Escherichia coli* to 87.5% in *Pseudomonas aeruginosa* with no difference in prevalence among patients on cotrimoxazole prophylaxis and those not on it. The high resistance to cotrimoxazole could be attributed to the excessive use of the drug for prophylactic purposes in HIV positive patients. In a South African study, 85.7% of *E. coli* isolated from HIV infected children was resistant to cotrimoxazole [20] while another South African study showed a significant increase in resistance to cotrimoxazole by organisms from 1999 to 2002 [21]. The World Health Organization noted that although cotrimoxazole is a broad spectrum antibiotic, increased use of the drug for prophylactic purposes would spur development of drug resistance which could ultimately render the drug ineffective when applied to common community pathogens [22, 23].

This current study found that the essential oils were made up of 68 compounds and the main components were nonanal (10.57%), phytol (6.40%), bicyclo[4.4.0]dec-1-ene, 2-isopropyl-5-methyl-9-methylene (5.65%), 4-tert-Butylcatechol, dimethyl ether (4.63%), 2-hexanol (2.95%), 5,9-Undecadien-2-one, 6,10-dimethyl- (2.91%) and naphthalene, 1,2,4a,5,6,8a-hexahydro-4,7-dimethyl-1-(1-methylethyl)- (2.91%) as shown in Table 3. The findings of this study are different from those of a similar study done by Boukhebti et al., (2015) in Algeria in which the essential oils were composed of 38 components. In the study palmitic acid (14.71%), z-nerolidol (9.45%), octacosane (6.32%), ceryophyllene oxide (4.77%), tetracosane (4.06%) and 4-hydroxy-4-methy-2-pentane (4.04%) were the main components of the essential oils [24].

A study by Upadhyay (2014) in India reported *O. europaea* plants to contain 24 compounds with the main components being 2-propanone (8.80%), 2,4imidazolidinedione (14.92%) and z-(13,14,-Epoxy) tetra dec-11-en-1-ol acetate (7.77%) [25]. Different studies in Tunisia reported differences in phytochemical composition of *Olea* extracts. Plant extracts have variations in total phenols, colour, composition of polysaturated fatty acids and water content [26–28]. The variation in chemical composition is attributed to differences in climatic conditions under which the plant grew or cultivation conditions such as irrigation and the ripening stage of the plant [26, 29]. Water content affects activity of enzymes responsible for synthesis of phytochemicals like phenols and increased water supply to plants increases the content of phenolic components, alcohols and esters such as (E)-2-hexanol [26].

Extracts from *O. europaea* had antimicrobial activity against GBS isolates from both South Africa and Namibia as shown in Table 4 and other studies have shown that *O. europaea* has antimicrobial activity against other bacteria although the extracts do not have broad spectrum antimicrobial activity [9]. While *Olea* has been shown to have antimicrobial properties in several countries including Spain, Israel, Palestine, plants in different geographical locations exhibit varying phytochemical constitutions therefore resulting in different properties and thus ethno botanical uses of the same plant [9, 30].

**Table 4:** Screening for antimicrobial activities of plant extracts against GBS isolates

Extract (Concentration)	Zone of inhibition on MH (Mean diameter)
Essential oil (300 mg/ml)	Present 25 mm
Leaf extract (0.5 mg/ml)	Present 28 mm
5 μg Ciprofloxacin (Positive control)	Present 35 mm
10% DMSO (Negative control)	Not present 0 mm

*O. europaea* essential oils showed bactericidal activity against GBS at 33.3 mg/mL as shown in Table 5 and studies have shown that essential oils have antimicrobial activity against a wide range of bacteria [9, 10]. Extracts from the leaves exhibited bactericidal activity at very low concentration (0.02 mg/mL) as presented in Table 6 and the antimicrobial activity could be attributed to phytochemical components present in the *Olea* plant extract such as flavonoids, iridoids, secoiridoids, biophenols and benzoic acid derivatives [1, 3, 9].

**Table 5:** MIC for essential oils against GBS isolates

Concentration (mg/mL)													
	0.05			0.4			3.7			33.3			MIC
Isolates	A	B	C	A	B	C	A	B	C	A	B	C	Essential oils
GBS in broth	+	+	–	+	+	–	+	+	–	–	+	–	33.3
GBS on MH	+	+	–	+	+	–	+	+	–	–	+	–	33.3

Key: A = Essential oil, B = 10% DMSO, C = Ciprofloxacin, + = Growth, − = no growth

Essential oils showed antibacterial activity to GBS at an MIC of 33.3 mg/mL and had bactericidal effect on the isolates at that concentration

**Table 6:** MIC for leaf extracts against GBS isolates

Concentration (mg/mL)													
	0.0008			0.004			0.02			0.1			MIC
Isolates	A	B	C	A	B	C	A	B	C	A	B	C	Leaf extracts
GBS in broth	+	+	–	+	+	–	–	+	–	–	+	–	0.02
GBS on MH	+	+	–	+	+	–	–	+	–	–	+	–	0.02

Key: A = Leaf extracts, B = 10% DMSO, C = Ciprofloxacin, + = Growth, − = no growth

Leaf extracts showed antibacterial activity to GBS at an MIC of 0.02 mg/mL and had bactericidal effect on the isolates at that concentration

## Conclusions

GBS isolates from South Africa and Namibia did not exhibit a lot of resistance to antibiotics and they showed sensitivity to *Olea europaea* essential oils and plant extracts. With the increasing resistance of bacteria to antibiotics in the world, *Olea* plant extracts and essential oils could potent as alternative therapy to GBS infection.

## Abbreviations

AEMREG: Applied and Environmental Microbiology Research Group
AIDS: Acquired immunodeficiency syndrome
ATCC: American Type Culture Collection
CDC: Centers for Disease Control and Prevention
CLSI: Clinical Laboratory Standards Institute
DMSO: Dimethyl Sulfoxide
GBS: Group B Streptococcus
GC-MS: Gas Chromatography-Mass Spectrometry
GMRDC: Govan Mbeki Research & Development Centre
HIV: Human immunodeficiency virus
IAP: Intrapartum Antibiotic prophylaxis
MH: Meuller hinton
MIC: Minimum inhibitory concentration
NCRST: National Commission of Research Science and Technology
NRF: National Research Fund
TBE: Tris/Borate/EDTA
UFH: University of Fort Hare
USA: United States of America
WHO: World Health Organization
